# The modified SAVE score: predicting survival using urgent veno-arterial extracorporeal membrane oxygenation within 24 hours of arrival at the emergency department

**DOI:** 10.1186/s13054-016-1520-1

**Published:** 2016-10-22

**Authors:** Wei-Cheng Chen, Kuo-Yang Huang, Chih-Wei Yao, Cing-Feng Wu, Shinn-Jye Liang, Chia-Hsiang Li, Chih-Yeh Tu, Hung-Jen Chen

**Affiliations:** 1 Division of Pulmonary and Critical Care, Department of Internal Medicine, China Medical University Hospital, No. 2, Yude Road, North District, Taichung City, 40402 Taiwan; 2 Division of Chest Medicine, Department of Internal Medicine, Yuanlin Christian Hospital, Changhua, Taiwan; 3 Division of Chest Medicine, Department of Internal Medicine, Everan Hospital, Taichung, Taiwan; 4 Division of Cardiovascular Surgery, Department of Surgery, China Medical University Hospital, Taichung, Taiwan; 5 Department of Respiratory Therapy, China Medical University, Taichung, Taiwan

**Keywords:** Extracorporeal membrane oxygenation, Outcome, Emergency, Acute respiratory distress syndrome, Cardiac failure, Critical care

## Abstract

**Background:**

Although many risk models have been tested in patients who undergo extracorporeal membrane oxygenation, few have been assessed for patients who received veno-arterial extracorporeal membrane oxygenation (VA-ECMO) support in the emergency department (ED). This study aimed to successfully predict outcomes of patients with cardiac or noncardiac failure who received VA-ECMO in the ED within 24 hours of arrival at the ED.

**Method:**

This retrospective, observational cohort study included 154 patients, who were classified as cardiac (*n* = 127) and noncardiac (*n* = 27) patients and received VA-ECMO within 24 hours after arrival at the China Medical University Hospital ED in Taiwan between January 2009 and September 2014. We recorded mechanical ventilation settings, arterial blood gases, laboratory parameters including plasma lactate level, requirement of catecholamines, and risk scores at time of ECMO initiation. ECMO and mechanical ventilation support duration, length of stay in the hospital, and 90-day mortality data were also examined.

**Results:**

The overall mortality rate was 64.9 %. We used “survival after veno-arterial ECMO (SAVE)” scores to assess survival prediction in survival and nonsurvival groups, which was statistically different (–3.2 vs. –8.3, *p* <0.001). According to multivariate Cox proportional regression of survival, lactate (hazard ratio [HR] = 1.01, 95 % confidence interval [CI], 1.01–1.01, *p* <0.001) and SAVE score (HR = 0.92, [95 % CI, 0.88–0.96], *p* = 0.001) were independent predictors of outcome. Excellent discrimination (area under curve (AUC) = 0.843) was observed when lactate and SAVE score were combined, which we referred to as “the modified SAVE score.”

**Conclusions:**

Modified SAVE scores improved outcome prediction for patients who underwent urgent VA-ECMO in the ED.

## Background

It is essential to make a timely and accurate diagnosis for patients who present to the emergency department (ED) with acute respiratory failure or circulatory failure, and an adequate treatment plan should be established based on the initial diagnosis. For example, dyspnea with respiratory failure is frequent in patients admitted to the ED, and rapid deterioration, including sudden cardiac arrest, may develop. However, immediate and accurate discrimination of heart conditions from other causes of dyspnea remains a clinical challenge, and approximately 20 % of patients are misdiagnosed [1]. Due to this, extracorporeal membrane oxygenation (ECMO), including veno-arterial (VA) and veno-venous (VV) methods, can temporarily stabilize such patients using mechanical devices and provide sufficient time to obtain a definitive diagnosis [2]. VA-ECMO enhances the survival rate of patients experiencing refractory cardiogenic shock [3], and VV-ECMO has been proposed as a possible rescue therapy for patients with severe acute respiratory failure that is refractory to conventional therapy [4–6]. Moreover, the combination of cardiopulmonary resuscitation (CPR) and VA-ECMO, which is known as E-CPR, yields promising results, compared with those of conventional CPR (C-CPR), with regard to patients experiencing in-hospital cardiac arrest (IHCA) or out-of-hospital cardiac arrest (OHCA) [7].

Despite the increasing experience with ECMO and technical improvements, the mortality of patients who receive ECMO remains high [2, 4, 8–10]. Furthermore, extended use of ECMO, with its associated requirements for a well-skilled ECMO team and resources may raise hospital costs [6]. Therefore, early identification of mortality risk factors is needed. Schmidt et al. recently developed the “predicting death for severe area under curve on VV-ECMO (PRESERVE)” score and the “respiratory extracorporeal membrane oxygenation survival prediction (RESP)” score to predict mortality of patients experiencing acute respiratory distress syndrome (ARDS) [11, 12]. For cardiac ECMO, lactate, bilirubin, and creatinine levels as well as postcardiotomy status have been shown to exhibit significant predictive value [13, 14]. Schmidt et al. also developed the “Survival After Veno-arterial ECMO (SAVE)” score to predict survival after VA-ECMO for refractory cardiogenic shock using 12 pre-ECMO parameters: age, weight, diagnosis, chronic renal failure, acute pre-ECMO organ failure, peak inspiratory pressure, duration of intubation, pre-ECMO cardiac arrest, pulse pressure before ECMO, diastolic pressure before ECMO, HCO_3_ level before ECMO and a constant value to add to all calculations of SAVE score [15]. Different predictive parameters are used for E-CPR patients [16].

Currently, not much is known by the time patients are urgently indicated for ECMO support in the ED. Although a number of risk models have been tested in patients who receive ECMO, few models have been tested in a patient population that specifically received ECMO support in the ED [17]. In this situation, we want to know if pre-ECMO scores could be used to assess patients receiving VA-ECMO support, which is the most frequently used ECMO mode in the ED.

This study was designed to assess the survival rate of patients with both cardiac and noncardiac conditions who received VA-ECMO in the ED within 24 hours of admission to the ED at our institution. In addition, this study seeks to determine if the SAVE score, which was created to predict survival after VA-ECMO for refractory cardiogenic shock, was applicable to patients who received VA-ECMO in the ED and identify the risk predictors for 90-day mortality.

## Methods

### Identified patients

A retrospective review of a prospective database indicated that 188 patients received ECMO within 24 hours after arrival at the ED between January 2009 and September 2014 at the China Medical University Hospital in Taichung, Taiwan. China Medical University Hospital is a medical referral center that has 130 intensive care unit beds and received 152,560 patient visits in the ED in 2014. After the exclusion of 16 patients who received VV-ECMO, nine patients with incomplete data, and nine patients who received ECMO due to operation, the study population consisted of 154 patients.

### Daily routine of ECMO configurations

The criteria used for evaluating the need for VA-ECMO to treat refractory circulatory failure was assessed as previously described [18]. The decision to initiate ECMO was made by the emergency physician in charge in conjunction with the ECMO team. The ECMO team was available at all times and included a perfusionist, an anesthesiologist, and a cardiovascular surgeon. After consultation, the ECMO team evaluated whether or not the patient was indicated for ECMO, and if necessary, the ECMO device was transported to the ED within 15 min.

### Outcome variables

Clinical details were retrospectively extracted from a prospective record of the ECMO database. Demographics, comorbidities, and reasons for ECMO were collected. Patients that required chest compressions in the ED were defined as having IHCA, while patients that required chest compressions prior to hospital arrival were defined as having OHCA. The “cardiac cause” was specified as pumping failure, and the “noncardiac cause” was defined as hypoxemic respiratory failure with or without circulation failure, most of which was due to ARDS.

Mechanical ventilation settings, arterial blood gases, and laboratory parameters, including plasma lactate level, requirement of catecholamines, and risk scores (sequential organ failure assessment [SOFA] and SAVE scores), were recorded at the time of ECMO initiation. ECMO and mechanical ventilation support duration, length of hospital stay, and 90-day mortality data were also extracted. Furthermore, the assessment of functional neurologic outcome of survivors with cerebral performance category (CPC) [19] was recorded. The five categories are: CPC 1, good cerebral performance (normal life); CPC 2, moderate cerebral disability (disabled but independent); CPC 3, severe cerebral disability (conscious but disabled and dependent); CPC 4, coma/vegetative state (unconscious); CPC 5, brain death.

### Statistical analyses

Continuous variables are expressed as median and interquartile ranges (25^th^ and 75^th^ percentile) and categorical variables are expressed as percentages. The chi-squared and Mann-Whitney *U* test were used to detect differences between survivors and nonsurvivors for categorical and continuous variables, respectively. Kaplan-Meier curves and log-rank tests were used to assess time to death from the date of ECMO initiation to the 90-day survival for groups stratified according to SAVE score.

Cox proportional hazards models were used to evaluate the univariate and multivariate hazard ratios (HR) for risk predictors of 90-day mortality. A candidate variable with a univariate *p* ≤0.05 was retained in the multivariable model. Variables included in the multivariable model were analyzed by correlation matrix to detect multicollinearity. When variables were associated with each other (i.e., pH and lactate), only one was included in the analyses. For practical purposes, continuous variables were converted into categories for incorporation in the final logistic regression model. The beta-coefficients were rounded to the nearest integer by dividing by the smallest coefficient.

The predictive accuracy of the mortality by variant index pre-ECMO was assessed using the area under the receiver operating characteristic (ROC) curve. *p* <0.05 was considered statistically significant. All statistical analyses were performed using SPSS software version 17.0 (SPSS Inc., Chicago, IL, USA) or MedCalc version 15.6.1 (MedCalc, Mariakerke, Belgium).

## Results

**Fig. 1:**
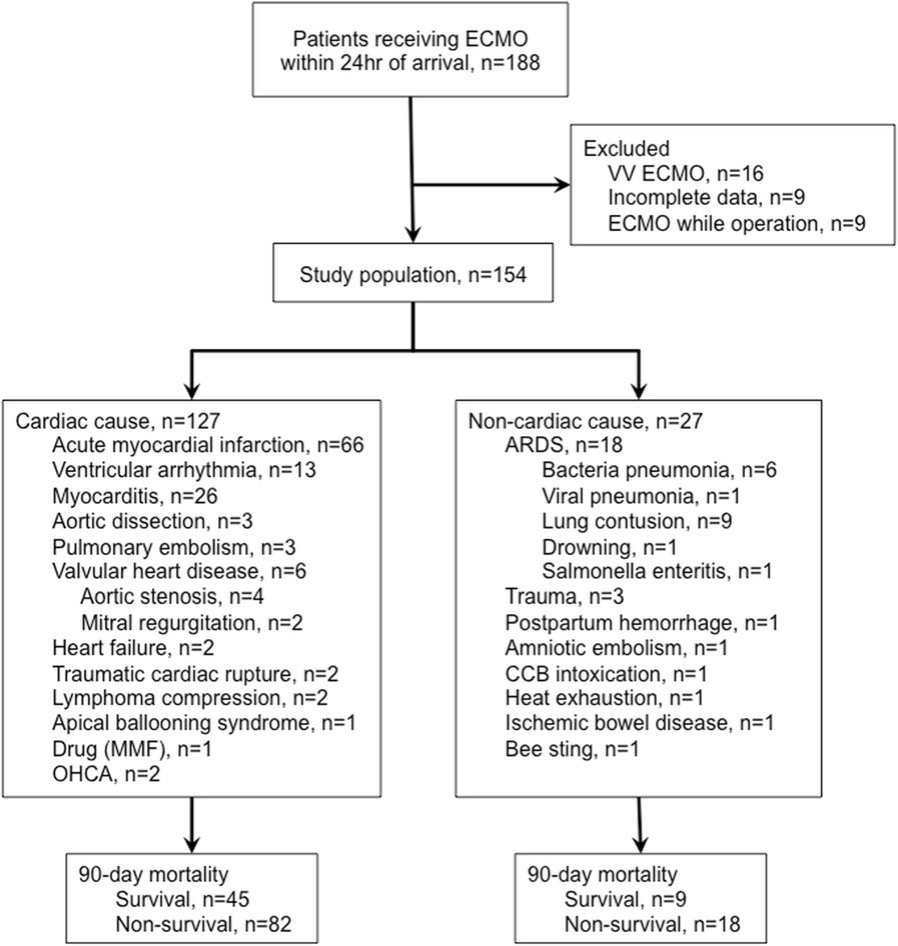
Flowchart of enrolled subjects. A total of 154 patients received urgent extracorporeal membrane oxygenation (ECMO) within 24 hours after arrival at the emergency department (ED), including 127 patients with cardiac causes and 27 patients with noncardiac causes. *ARDS* acute respiratory distress syndrome, *CCB* calcium channel blocker, *MMF* mycophenolate mofetil, *OHCA* out-of-hospital cardiac arrest, *V-A mode* veno-arterial mode, *V-V mode* veno-venous mode

During the study period, we assessed 154 patients who were an average age of 51.7 years (range, 38–69 years) and of which 72.1 % were male who received ECMO support within 24 hours of arrival at the ED. One hundred twenty-seven patients received ECMO support due to cardiac causes, and 27 patients received support due to noncardiac causes (Fig. 1). A large proportion of patients with noncardiac causes were admitted because of ARDS. There were 54 patients who were ultimately discharged, and the overall mortality rate was 64.9 %. A total of 64.6 % of patients with cardiac cause and 66.7 % with noncardiac cause died. Among the 54 survivors, 18 of 54 (33.3 %) had a CPC of 1; 13 of 54 (24.0 %) had a CPC of 2; 12 of 54 (22.2 %) had a CPC of 3; and 11 of 54 (20.3 %) had a CPC of 4.

Baseline characteristics and comparative results of the patients who survived and those who did not are shown in Table 1. Before ECMO initiation, survivors were younger in age (43.1 vs. 56.4 years, *p* <0.001) and had less acidosis (pH 7.20 vs. 7.06, *p* <0.001); less hypercapnia (PaCO_2_ 40.1 vs. 51.3 mmHg, *p* = 0.007); higher bicarbonate levels (HCO_3_^−^ 15.6 vs. 12.7 mmol/L, *p* = 0.001), lower lactate levels (64.9 vs. 115.2 mg/dL, *p* <0.001), higher platelet counts (216.8 vs. 184.9 × 10^3^/μL, *p* = 0.036), and lower severity scores (SOFA score 8.7 vs. 10.5, *p* = 0.004). Additionally, the proportion of OHCA was lower in the survival group (22.2 % vs. 41.0 %, *p* = 0.031). Sex, Charlson score, body mass index, PaO_2_/FiO_2_, plateau pressure, and other laboratory data were not significantly different among survivors and nonsurvivors. We assessed SAVE scores for survival prediction in the two groups, which were statistically different (–3.2 vs. –8.3, *p* <0.001).

**Table 1:** Intensive care scores and mechanical ventilation variables at the time of ICU admission

	All patients (*N* = 154)	Survivors (*N* = 54)	Nonsurvivors (*N* = 100)	*p* value
Age (yr)	51.7 (38.0–69.0)	43.1 (28.0–62.0)	56.4 (42.5–70.0)	<0.001
Male (n, %)	111 (72.1)	35 (64.8)	76 (76)	0.103
Charlson score	0.8 (0–1.0)	0.6 (0–1.0)	1.0 (0–1.0)	0.18
Body mass index >30 kg/m^2^ (n, %)	23 (14.9)	7 (13.0)	16 (16.0)	0.79
Immunocompromised (n, %)	19 (12.3)	5 (9.3)	14 (14.0)	0.55
Pre-ECMO blood gases				
pH	7.11 (6.97–7.24)	7.20 (7.07–7.31)	7.06 (6.92–7.18)	<0.001
PaO_2_/FiO_2_ (mmHg)	144.9 (60.0–209.0)	146.7 (62.0–190.0)	144.0 (52.5–212.5)	0.21
PaCO_2_ (mmHg)	47.3 (32.0–59.0)	40.1 (31.0–51.0)	51.3 (32.5–67.5)	0.007
HCO_3_^-^ (mmol/L)	13.7 (10.2–17.7)	15.6 (13.2–18.4)	12.7 (9.1–16.6)	0.001
Mechanical ventilation				
Plateau pressure (cmH_2_O)	29.5 (27.0–32.0)	28.5 (26.0–30.0)	30.0 (27.0–33.0)	0.06
Laboratory parameters				
Lactate (mg/dL)	97.4 (56.0–125.1)	64.9 (40.0–75.0)	115.2 (75.3–144.4)	<0.001
Hct (%)	39.4 (35.0–45.0)	39.6 (35.7–44.2)	39.3 (34.3–45.4)	0.93
Platelet count (× 10^3^/μL)	196.1 (147.0–242.0)	216.8 (153.0–280.0)	184.9 (124.5–224.0)	0.036
Creatinine (mg/dL)	1.97 (1.09–1.95)	1.75 (0.98–1.90)	2.09 (1.14–2.06)	0.06
Bilirubin (mg/dL)	1.02 (0.50–0.91)	0.80 (0.50–0.81)	1.13 (0.50–0.93)	0.09
CRP	3.25 (0.31–2.15)	2.52 (0.22–1.21)	3.65 (0.33–3.28)	0.06
Vasopressors before ECMO (n, %)	134 (87.0)	26 (48.1)	81 (81.0)	0.21
Cardiac arrest before ECMO				
IHCA (n, %)	62 (40.3)	18 (33.3)	44 (44.0)	0.09
OHCA (n, %)	53 (34.4)	12 (22.2)	41 (41.0)	0.031
SOFA score	9.8 (7.0–13.0)	8.7 (6.0–12.0)	10.5 (8.0–13.0)	0.004
SAVE score	–6.5 (–10.0– –3.0)	–3.2 (–7.0–2.0)	–8.3 (–11.0– –5.0)	<0.001

*CRP* C-reactive protein, *ECMO* extracorporeal membrane oxygenation, *IHCA* in-hospital cardiac arrest, *OHCA* out-of-hospital cardiac arrest, *SAVE score* Survival After Veno-arterial ECMO score, *SOFA score* Sequential Organ Failure Assessment score, *V-A mode* veno-arterial mode, *V-V mode* veno-venous mode

**Fig. 2:**
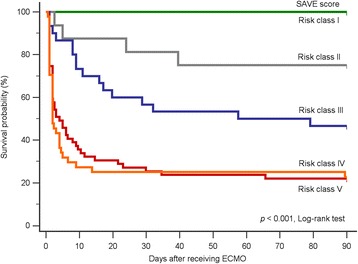
Kaplan-Meier survival curves according to different SAVE score classes. *SAVE score* Survival After Veno-arterial Extracorporeal membrane oxygenation score

Kaplan-Meier survival curves according to SAVE score classes are plotted in Fig. 2. The log-rank test showed significantly different risks between groups (*p* <0.001). However, it was challenging to discriminate survival probabilities with SAVE scores in more severe risk classes (class IV and V).

By univariate analysis, age, OHCA, pH, HCO_3_^−^, PaCO_2_, lactate, platelet count, and SOFA and SAVE scores were potential prognostic factors for 90-day mortality (Table 2). Multivariate Cox proportional hazards regression of survival was conducted using these variables, revealing SAVE score (HR = 0.92, [95 % CI, 0.88–0.96], *p* = 0.001) and lactate level (HR = 1.01, [95 % CI, 1.01–1.01], *p* <0.001) to be independent variables.

**Table 2:** Cox proportional hazards regression model analysis for prognostic factors of emergency ECMO use

Variables	Univariate analysis		Multivariate analysis	
	Hazard ratio (95 % CI)	*p* value	Hazard ratio (95 % CI)	*p* value
SAVE score	0.92 (0.90-0.95)	<0.001	0.92 (0.88–0.96)	0.001
SOFA score	1.11 (1.04–1.17)	0.001	0.96 (0.89–1.05)	0.37
Age (yr)	1.02 (1.01–1.03)	<0.001	NE^a^	
OHCA	1.77 (1.19–2.64)	0.005	1.13 (0.69–1.86)	0.62
Laboratory findings				
PaCO_2_ (mmHg)	1.02 (1.01–1.03)	0.001	1.00 (0.99–1.01)	0.93
HCO_3_^-^ (mmol/L)	0.94 (0.91–0.97)	0.001	NE^a^	
Lactate (mg/dL)	1.01 (1.01–1.01)	<0.001	1.01 (1.01–1.01)	<0.001
Platelet (x10^3^/μL)	1.00 (0.99–1.00)	0.007	1.00 (1.00–1.00)	0.11
pH	0.07 (0.02–0.21)	<0.001	NE^b^	

*CI* confidence interval, *ECMO* extracorporeal membrane oxygenation, *IHCA* in-hospital cardiac arrest, *OHCA* out-of-hospital cardiac arrest, *SAVE score* Survival After Veno-arterial ECMO score, *SOFA score* Sequential Organ Failure Assessment score,

^a^Not entered into the multivariate analysis because age and HCO_3_^-^ are involved in SAVE score

^b^Not entered into the multivariate analysis because of pH and lactate with high collinearity

**Fig. 3:**
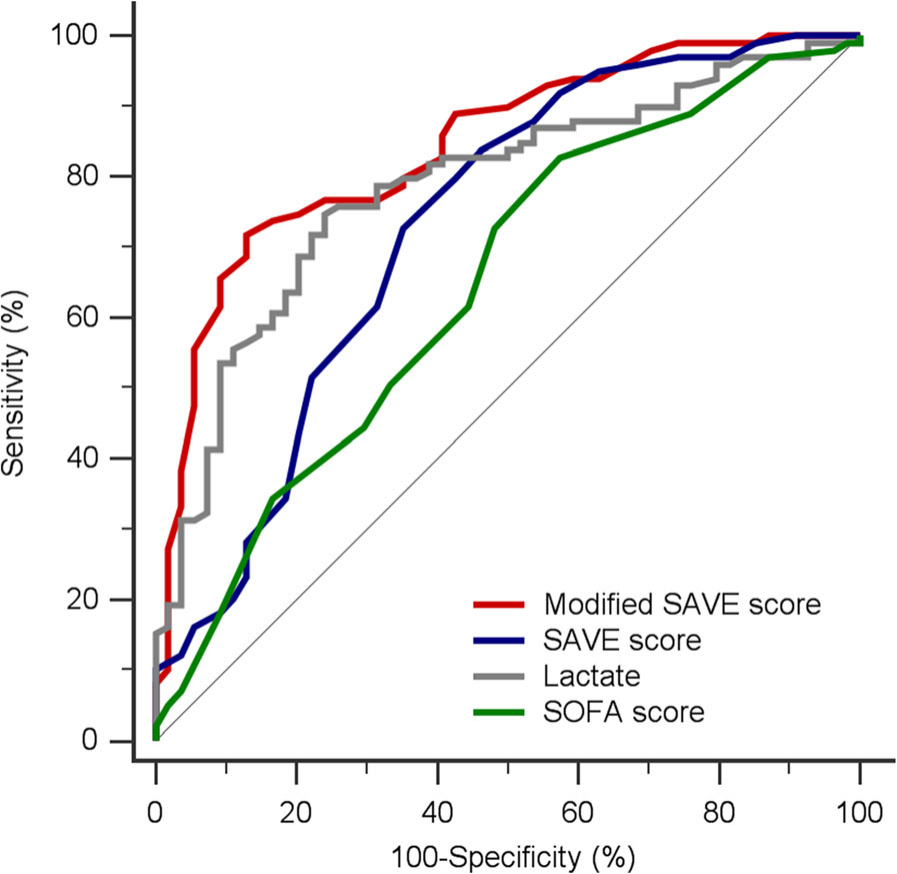
ROC curves for predicting 90-day mortality in patients who receive emergency ECMO according to different variables. Various AUC values were seen when predicting 90-day mortality: SOFA (*green line*) = 0.65, lactate (*gray line*) = 0.79, SAVE score (*blue line*) = 0.73, and “the modified SAVE score” (combination of lactate and SAVE score) (*red line*) = 0.84. *AUC* area under the curve, *ROC curve* receiver operating characteristic curve, *SAVE score* Survival After Veno-arterial Extracorporeal membrane oxygenation score, *SOFA score* Sequential Organ Failure Assessment score

In the ROC curves used to predict 90-day mortality, the SOFA score showed limited discrimination (area under curve, AUC of SOFA score = 0.65), while lactate level and SAVE score showed acceptable discrimination (AUC for lactate = 0.79; AUC for SAVE score = 0.73) (Fig. 3).

Because lactate and SAVE score were both statistically significant mortality predictors in the Cox proportional hazards regression model and both showed acceptable discrimination with ROC curves, we developed a new model that incorporated lactate data into the SAVE score. Lactate level was dichotomized into groups of patients with levels <75 mg/dL and ≥75 mg/dL based on data that has maximal Youden’s index. The odds ratios (ORs) for both SAVE score (OR = 1.17, 95 % confidence interval [CI]: 1.08–1.26, *p* <0.001) and lactate level (OR = 8.74, 95 % CI: 3.81–20.06, *p* <0.001) were increased in the logistic regression model (Table 3). The regression coefficients for these two predictors were rounded to the nearest integer risk score and summed to calculate the modified SAVE scores in Table 3. For example, if the patient had a SAVE score of 5 and lactate level of 60 mg/dl, his modified SAVE score would be 5 + 15 = 20.

**Table 3:** Logistic regression model analysis and coefficients for SAVE scores and categorical lactate quantities for survival prediction

Variables	Coefficient	Modified SAVE score	Odds ratio (95 % CI)	*p* value
SAVE score	0.16	SAVE score	1.17 (1.08–1.26)	<0.001
Lactate (mg/dL)				
< 75	2.17	15	8.74 (3.81–20.06)	<0.001
≥ 75		0		

*CI* confidence interval, *SAVE score* Survival After Veno-arterial ECMO score,

Modified SAVE scores ranged from –35 to 32 and were divided into five risk classes, referred to as class I (score >10), class II (score 1 to 10), class III (score -4 to 0), class IV (score –10 to –5) and class V (score < -10). The predicted 90-day mortality of patients with these modified SAVE scores were 21, 48, 68, 90 and 96 %, respectively. In the ROC curves used to predict 90-day mortality, excellent discrimination (AUC = 0.84) was seen for the modified SAVE score (Fig. 3).

## Discussion

To the best of our knowledge, this is the first and largest study focusing on patients who required VA-ECMO to treat circulatory failure within 24 hours after arrival at the ED. We tested the efficacy of the SAVE score, which was originally created for patients receiving VA-ECMO due to refractory cardiogenic shock, and showed that SAVE scores were helpful when used to assess patients receiving VA-ECMO in the ED. In this study, we also created a strong predictive survival model specifically for patients receiving VA-ECMO using the combination of blood lactate level and SAVE score, which we called the modified SAVE score. This score resulted in best discrimination in the mortality prediction model.

Although ECMO can provide total circulatory support and fully correct gas-exchange abnormalities, the mortality of patients undergoing severe cardiogenic shock or ARDS who receive urgent ECMO remains high. In the CESAR trial, in which 180 patients with severe ARDS were randomly assigned to receive ECMO, the mortality in a 6-month period was 37 % [6]. For cardiac ECMO patients, mortality rates reached 57–80 % [13, 14]. In our 154 patients who underwent VA-ECMO within 24 hours of arrival at the ED, including 127 and 27 patients with cardiac and noncardiac causes, respectively, the mortality rates were 64.6 % and 66.7 %, respectively. The mortality rates were higher than those in the Extracorporeal Life Support Organization (ELSO) registry report (60 % for cardiac ECMO and 44 % for respiratory ECMO) [20]. The high percentage (74.7 %) of cardiac arrest before ECMO initiation in our patient population may explain the differences in survival. Furthermore, noncardiac patients who received VA-ECMO in the ED usually were experiencing shock and/or cardiac arrest, which have worse patient outcomes. This may explain the high mortality rate of noncardiac patients in our study.

To avoid unnecessary use of ECMO, which might unnecessarily consume resources and expose patients to possible ECMO complications, thorough consideration must be used to identify the appropriate candidates for ECMO support. Hence, a number of studies have established risk scores to predict mortality in patients receiving ECMO. The well-known PRESERVE and RESP scores designed by Schmidt et al. were designed for patients with ARDS who required VV-ECMO support [11, 12, 21]. Recently, Schmidt et al. created the SAVE score, which yielded an AUC value of 0.68 for patients supported by VA-ECMO for refractory cardiogenic shock [15]. Since specifically analyzed patients receiving VA-ECMO, we used the SAVE score rather than PRESERVE or RESP scores in our cohort to predict 90-day mortality and showed an acceptable AUC value of 0.73. Additionally, SAVE score was found to be an independent variable in the Cox proportional hazards regression model. In our cohort, Kaplan-Meier survival analysis by log-rank test for SAVE score classes revealed significantly different survival times among stratified patients. However, it was challenging to totally discriminate survival within various SAVE score classes (Fig. 2). Otherwise, by multivariate Cox proportional hazards regression analysis, high pre-ECMO blood lactate levels combined and low SAVE scores emerged as risk factors of mortality.

Several studies have shown a close association between high blood pre-ECMO lactate level and mortality in patients receiving ECMO for cardiac causes [22, 23]. Shorter CPR durations during E-CPR for IHCA or OHCA lead to lower titers of lactate, higher survival rates, and better neurologic outcomes [24]. However, in our series, OHCA had no significant predictive value by multivariate analysis when combined with lactate. Otherwise, our study showed that increased lactate levels gradually increased the hazard ratio, and with every increase of 1 mg/dL, the mortality risk increased by 1 %. Lactate levels discriminated survivors from nonsurvivors with an AUC of 0.79. When lactate was combined with SAVE score, the mortality prediction achieved the highest AUC of 0.84. Therefore, we developed an amalgamation between these two variables called the modified SAVE score. Using the modified SAVE score (score -35 to 32), we predicted survival of patients receiving VA-ECMO support in the ED to reveal predicted 90-day mortality ranging from 21 to 96 %.

This study is strengthened by the large number of included patients. However, certain limitations should be considered. First, it is a single-center retrospective study, which could restrict the translation of our results to other hospitals. Second, although we included only patients receiving VA-ECMO support in the ED, the underlying diseases within our cohort were diverse. Nevertheless, it stands that the modified SAVE score could be applied to patients with circulatory failure even if a definitive diagnosis could not be obtained in time, which is not uncommon in the ED. Third, we did not have enough patients to internally validate the model. Further research is needed to better assess the external validity of the modified SAVE score in a large independent cohort.

## Conclusions

In conclusion, our study indicates that the combination of blood lactate level and SAVE score, termed the modified SAVE score, results in improved discrimination of outcome predictions for patients who receive VA-ECMO support in the ED. The modified SAVE score will help emergency or critical care physicians evaluate and treat heterogeneous patients who were indicated for VA-ECMO support with little information concerning the cause of the critical condition.

## Abbreviations

ARDS: acute respiratory distress syndrome
AUC: area under curve
C-CPR: conventional cardiopulmonary resuscitation
CI: confidence interval
CPC: cerebral performance category
CPR: cardiopulmonary resuscitation
ED: emergency department
ELSO: Extracorporeal Life Support Organization
HR: hazard ratio
IHCA: in-hospital cardiac arrest
OHCA: out-of-hospital cardiac arrest
OR: odds ratio
PRESERVE: predicting death for severe area under curve on veno-venous extracorporeal membrane oxygenation
RESP: respiratory extracorporeal membrane oxygenation survival prediction
ROC: receiver operating characteristic
SAVE: survival after veno-arterial extracorporeal membrane oxygenation
SOFA: sequential organ failure assessment, VA-ECMO, veno-arterial extracorporeal membrane oxygenation
VV-ECMO: veno-venous extracorporeal membrane oxygenation
